# Parental Socialization Styles: The Contribution of Paternal and Maternal Affect/Communication and Strictness to Family Socialization Style

**DOI:** 10.3390/ijerph16122204

**Published:** 2019-06-21

**Authors:** Inge Axpe, Arantzazu Rodríguez-Fernández, Eider Goñi, Iratxe Antonio-Agirre

**Affiliations:** 1 Department of Developmental and Educational Psychology, University of the Basque Country (UPV/EHU), 48940 Leioa, Spain; 2 Department of Developmental and Educational Psychology, University of the Basque Country (UPV/EHU), 01006 Vitoria-Gasteiz, Spain; arantzazu.rodriguez@ehu.eus (A.R.-F.); eider.goni@ehu.eus (E.G.); iratxe.antonio@ehu.eus (I.A.-A.)

**Keywords:** family socialization style, affect/communication, strictness, adolescence

## Abstract

The aim of this study is two-fold: (a) to determine the general degree of family affect/communication and strictness by examining the combination of the two classical dimensions of mother parenting style: affect/communication and strictness, and (b) to analyze the impact of both parents’ affect and strictness on the family style, thereby exploring the specific contribution made by each parent’s style and dimension. Participants were 1190 Spanish students, 47.1% boys and 52.3% girls (M = 14.68; SD = 1.76). The *Affect Scale* (EA-H) and the *Rules and Demandingness Scale* (ENE-H) (both by Fuentes, Motrico, and Bersabé, 1999) were used. Structural equation models (SEMs) were extracted using the EQS program. The results reveal that it is not the father’s and the mother’s parenting style combined, but rather the combination of maternal and paternal affect/communication, and maternal and paternal strictness which generates one perception of family affect and another of family strictness. The results also indicated that the weight of both dimensions varies in accordance with the parent’s gender, with maternal dimensions playing a more important role in family socialization style.

## 1. Introduction

### 1.1. Parental Socialization Styles and their Dimensions

From childhood onwards, the family is the foremost context for socialization and individual development, and parents represent one of the most powerful influences in their children’s lives [1,2]. Far from being restricted to childhood, however, this influence continues throughout individuals’ entire lives [3,4], becoming particularly relevant in moments of change, such as adolescence, which is now considered the most complex period of the life cycle due to the multiple simultaneous challenges faced by young people during the teenage years [5].

Traditionally, research into the way in which parents bring up and socialize their children and the consequences of their different practices for children’s adjustment and wellbeing has focused mainly on parental socialization styles. Although several different models have been proposed since the initial publication of studies in this field [6], the most commonly-used is that resulting from the combination of two underlying dimensions which have guided the study of parenting for decades [7,8]: responsiveness and demandingness [9,10]. Both refer to patterns of parental practices that are grouped into these two central dimensions considered theoretically [10] and empirically [11,12] as orthogonal (independent) dimensions. The combination of both orthogonal dimensions results in a quadrant of four parental styles [10,12]: the *authoritative* or *democratic* and the *permissive* or *indulgent* styles, characterized by high responsiveness and high and low demandingness (respectively), and the *authoritarian* and the *neglectful* styles, characterized by low responsiveness and high and low demandingness, respectively [13].

The responsiveness dimension, also termed warmth, affect, or acceptance [14], has generated a large volume of research attesting to its importance in offspring’s positive development, flourishing, and emotional development (e.g., self-esteem, subjective well-being, lower risk of behavioral problems, mood disorders [13,15,16,17], or drug consumption [18]). This relation with positive adjustment has been observed all the way to adulthood across many different cultures [19,20]. 

The consensus regarding the demandingness dimension, often defined as control, is not as broad [21], perhaps due to the variety of different conceptions that have been proposed [22] since the initial pioneering studies [9,13]. For example, demandingness has been found to be positive when understood as behavioral control and supervision of adolescents’ conduct [22,23], although not when it takes the form of coercive and psychological control [1,14,24,25]. The general trend is to consider psychological control as being detrimental to children’s development, while behavioral control is only harmful if it becomes coercive [9] and adolescents perceive it as excessively invasive or affecting aspects they do not consider to come under the purview of parental authority [26]. 

In the present study, the two dimensions are encompassed under the terms *affect-communication* (for responsiveness) and *discipline* or *strictness* (for demandingness). Thus, the affect*-*communication dimension is similar to the classic responsiveness one, being characterized by emotional warmth and the provision of help and support in accordance with the child’s needs [9]. In other words, it reflects open communication and displays of care, affection, concern, and acceptance by parental figures [21]. Demandingness, on the other hand, is conceptualized in terms of strictness or firmness [27,28], a label adopted by recent studies [11,12,29,30,31] which refers to the rigidity with which parents exercise their authority [32] and impose rules and limits on their children’s behavior [4].

### 1.2. The Contribution of Parental Figures to the Establishment of Family Socialization Style

Parental socialization styles are defined more as an emotional context or climate than as a set of specific parenting practices [13], meaning that depending on said context, each parent’s individual practices (affect, communication, strictness, etc.) would have a different meaning for their child [33]. 

It is therefore important to take the styles and practices of both parents into consideration, since both contribute to the establishment of the family socialization style. Analyzing how the father’s and the mother’s practices combine with each other provides a more realistic insight into two-parent households and families [34]. Nevertheless, previous studies have rarely taken both parents (mother and father) into account [35,36], tending to place more emphasis on the maternal style than the paternal one [22,37], possibly because women have traditionally spent more time with their children than men [38,39], assuming greater responsibility for their care and upbringing [21,40]. 

When both parents have been taken into account, in most cases it was to determine their relative weight in, or contribution to, their children’s adjustment outcomes, with the results indicating that both parents play a significant role [24,25,26,41,42]. However, no consensus has yet been reached regarding which parent’s influence is stronger, with evidence existing in support of both the paternal [17,25,36,40,43,44,45] and the maternal contribution [27,46]. It is possible that this lack of convergence may be due, among other issues, to the type of variable analyzed, with some authors for example, arguing that maternal affect fosters children’s socioemotional development, while (a lack of) paternal affect results in behavioral problems [17].

Whatever the case, the practices of both parents are rarely analyzed in order to determine the family or global style established as a result of their interaction, which ultimately determines the socialization of the children living in the household. In the few studies which mention a "family style", participants are asked about their parents’ practices in a general, non-specific manner, with no distinction being made between mothers and fathers [3,47,48]. Alternatively, the score of a single parent is used [2,4] or family style is established on the basis of a mean score, simply combining the scores obtained by the father and the mother for each specific dimension [49]. 

On other occasions, family style is determined by comparing the mother’s and the father’s style [34,38,50,51,52] in order to assess the level of congruence or incongruence between them, without taking the dimensions underlying these styles into consideration [53].

### 1.3. The Contribution of Parenting Dimensions to the Establishment of Family Socialization Style

Just as studies which consider both the maternal and the paternal style do not seem to analyze their contribution to the family socialization style, nor do any of the studies found analyze the contribution of each parent’s practices to this same construct. In general, studies which consider the practices of both parents tend to observe their relationship with children’s outcomes separately, with few taking into account the possible combined or interaction effect of maternal and paternal parenting dimensions [25,34,53,54].

Studies which report dimension measures for both mothers and fathers have found that they tend to coincide and correlate positively with each other, although mothers are usually perceived as more affectionate, as well as exercising greater control and discipline than fathers [2,37,55,56,57]. This may be due to the fact that, despite social advances and the increase in parental co-responsibility, gender construction and social stereotypes still result in women continuing to be more involved than men in the care and upbringing of their children [37]. 

### 1.4. The Present Study

None of the studies cited above analyze the contribution made by each individual parent to the family style, through the exercise of their parenting practices or dimensions. However, given that only a combined vision of the practices of both parents [17,25,58], in the form of the family socialization style, will enable a fuller understanding of the climate and significance of said practices in the key socialization context that is the family, it is vital to explore the way in which both parenting dimensions (*affect-communication* and *strictness*) contribute to establishing this style.

It is therefore important to have separate measures of affect and strictness for each parent [24,59,60] and to explore further the relationship which exists between the different dimensions [1,61] in order to determine their combined effect [17,25,34] and calculate their relative weight in the establishment of the family socialization style or climate, as perceived by children. This is, therefore, the fundamental aim of the present study.

## 2. Materials and Method

### 2.1. Participants

A total of 1224 participants were recruited by random sampling, although following the elimination of those with unlikely response patterns (outliers) and those failing to complete the two questionnaires on paternal and maternal socialization styles (due to different reasons, e.g., having a deceased parent, not having a relationship with one of two parents, etc), the final sample comprised only 1190. Participants were aged between 12 and 17 years (M = 14.68; SD = 1.76) and were all students at 8 secondary schools (4 semi-private schools with 449 participants and 4 public ones with 741 participants) in the Spanish Autonomous Community of the Basque Country (ACBC). With regards to sex, 560 (47.1%) were boys and 622 (52.3%) were girls, and 1024 (86.1%) claimed to have a medium family socioeconomic and cultural level, 36 (3%) claimed to have a low level, and 101 (8.5%) had a high level.

In terms of living arrangements, 1010 (84.9%) lived with their nuclear or nuclear-extended family, 100 (8.75%) lived with the parent who had custody of them, and 21 (1.8%) lived alternatively with both parents, under a joint custody regime. Finally, 31 (2.8%) lived in step-families and 28 failed to provide any data about their family situation, although they did respond to the questionnaires for both parents.

### 2.2. Measures

Two questionnaires were used to assess parenting styles, one for each dimension (affect-communication and strictness). In both cases, the children’s version of the instrument was used. Participants responded to each item in accordance with their perception of the style employed by the parent in question (mother or father). Although both questionnaires comprise various subscales, in each one we chose that which corresponded to the most widely-accepted theory, which views responsiveness (or affect-communication) and demandingness (or strictness) [7,8,9,32] as dimensions which together make up the four quadrants of the parenting style grid [7,8]. In both questionnaires, respondents answered on a 5-point Likert-type scale (1 = never to 5 = always), with higher scores indicating higher levels in all dimensions.

#### 2.2.1. Affect-Communication Dimension

To measure each parent’s level of affect-communication, the subscale used was that of the same name within the *Affect Scale* (EA-H) [62]. This subscale is designed to assess children’s perceptions of their parents’ affect, communication, and interest in them. Although the affect-communication subscale comprises 10 items (e.g.: “He is affectionate to me”; “He/she comforts me when I am sad”; “He/she spends time talking to me”), rated on a 5-point scale from 1 = never to 5 = always, only 7 were used here (i2, i6, i8, i9, i11, i14, and i20). All had adequate indexes for the study, both as regards to the confirmatory factor analysis (CFA) for the subscale and in terms of its composite reliability and average variance extracted (AVE). The goodness-of-fit indicators for the data in the CFA were Chi-squared = 48.96; *p* < 0.05; Root Mean Square Error of Approximation (RMSEA) = 0.074; Comparative Fit Index (CFI) = 0.965; Normed Fit Index (NFI) = 0.952; Non Normed Fit Index (NNFI) = 0.947; Incremental Fit Index (IFI) = 0.965 for paternal affect-communication; and Chi-squared = 33.63; *p* < 0.05; RMSEA = 0.063; CFI = 0.967; NFI = 0.951; NNFI = 0.943; IFI = 0.968 for maternal affect-communication. The values for composite reliability and AVE were (respectively) 0.876 and 0.505 for the father and 0.852 and 0.485 for the mother. The reliability indexes were therefore similar to those reported by other studies [21], and can be considered indicative of adequate composite reliability [63].

#### 2.2.2. Strictness Dimension

To measure parents’ strictness, the *Strictness* subscale of the Rules and Demandingness questionnaire (ENE-H) [62] was used. Specifically, 7 out of the 10 items (e.g.: “He/She tries to control my life all the time?”; “He/She imposes very harsh punishments on me, so that I do not disobey again”; “He/She tells me that parents are always right”) contained in said subscale were used here (i2, i4, i9, i20, i23, i25, and i27), with responses given on the same Likert-type scale as that used in the affect-communication dimension. A CFA was conducted to verify the goodness-of-fit of the data and to determine the composite reliability and average variance extracted (AVE). In the case of both father’s (X^2^_(13)_ = 39.75; p < 0.05; RMSEA = 0.067; CFI = 0.954; NFI = 0.933; NNFI = 0.925; IFI = 0.954) and mother’s strictness (X^2^_(13)_ = 33.93; p > 0.05; RMSEA = 0.060; CFI = 0.953; NFI = 0.927; NNFI = 0.923; IFI = 0.953), the results of the CFA confirmed the suitability of the items used to measure the variable. The composite reliability values were also adequate, despite the average variance extracted being close to the cutoff point (0.50), since values under this threshold may be considered acceptable, providing the composite reliability coefficients are equal to or higher than 0.70 and the item-subscale correlation coefficients are equal to or higher than 0.40, both of which were true in this case [63]: composite reliability = 0.858 and AVE = 0.441 for father’s strictness and composite reliability = 0.826 and AVE = 0.471 for mother’s strictness.

### 2.3. Procedure

Approval for the study (M10_2015_076) was obtained from the Ethics Board for Research with Human Beings (CEISH-UPV/EHU) at the University of the Basque Country, which attests to the fact that the procedure respects the basic principles established by the American Psychological Association [64], including informed consent and right to information, protection of personal data and guarantees of confidentiality, non-remuneration, and the possibility of withdrawing from the study at any time. Once the study had been approved, several schools were randomly selected from the Basque Regional Government’s list of all the secondary schools in the Autonomous Community of the Basque Country. The schools were then informed of the research project and invited to participate. Whenever a school declined to participate, another was selected from the list using the same random procedure, although always respecting the proportional split between semi-private and public institutions. Only students whose parents/guardians signed an informed consent form participated in the study. All questionnaires were completed in class time under the supervision of members of the research team. All students in the same class completed the questionnaire at the same time, and the mean duration was thirty minutes. With the aim of mitigating the incidence of responses in keeping with the research hypothesis, the single-blind criterion was employed (i.e., students were unaware of the purpose of the study). Furthermore, both the confidentiality of the responses given, and the voluntary nature of the participation were guaranteed in order to reduce the effects of the social desirability bias as far as possible.

### 2.4. Analysis

Missing values (1.92%) were inferred using the expectation maximization (EM) algorithm and the Markov chain Monte Carlo (MCMC). Outliers (inconsistent or strange response patterns, or extreme responses) were analyzed, with 34 participants (2.8%) being eliminated as a result. No participant was eliminated from the study for failing to respond to the minimum number of items.

To test the fit of the structural model to the data and to carry out the different CFAs to verify the suitability of the items selected, the SEM method was used within the EQS program, version 6.1 (Multivariate Software, Encino, CA, USA) [65]. Since the data did not reach the multivariate normality level required for this type of methodology, and given that the Mardia coefficient obtained was higher than 25 (p < 0.01), the robust ML (Maximum Likelihood) estimation method was used at all times, combining robust goodness-of-fit indexes with Satorra–Bentler’s chi-squared and significance level [66].

The polychoric correlation matrices were also calculated. To determine the fit of the different models hypothesized, we tested them in accordance with the instructions proposed by experts, which suggest the use of the following combination of indicators [67,68]: Chi-squared, along with its associated probability, Root Mean Square Error of Approximation (RMSEA), Normed Fit Index (NFI), Incremental Fit Index (IFI), and Comparative Fit Index (CFI). While for the RMSEA values under 0.08 are considered acceptable, for all of the other indicators’ values must be at least 0.90. Finally, the Akaike Information Criterion (AIC) and Consistent Akaike Information Criterion (CAIC) indicators were calculated to compare the estimated models. In these indicators, lower values represent better fit due to the greater parsimony of the model.

## 3. Results

**Figure 1:**
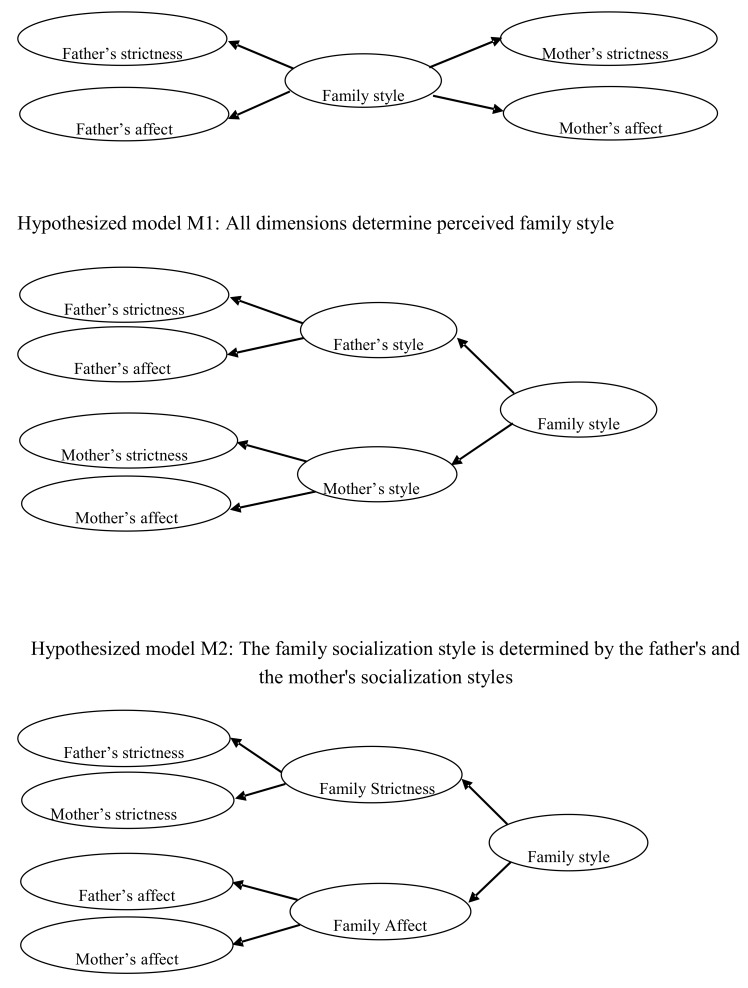
Hypothesized theoretical models.

To analyze the combination of the different dimensions which together make up parenting styles (both paternal and maternal) and their effect on family socialization style, three different structural regression models were empirically compared. The first model (M1) postulated that the four dimensions (mother’s affect-communication, father’s affect-communication, mother’s strictness, and father’s strictness) have a direct influence on family socialization style, as perceived by the child. The second model (M2) postulated that paternal dimensions (affect-communication and strictness) combine to form the paternal style, and that the maternal dimensions combine to form the maternal style, the child’s perception of both styles would then result in their perception of the family socialization style. The third and final model (M3) postulated that both affect-communication dimensions (father’s and mother’s) influence a variable called "family affect-communication", and both strictness dimensions (father’s and mother’s) influence a variable called "family strictness," both variables would then combine to give the family socialization style, as perceived by the child (see Figure 1).

Firstly, the suitability of the measurement model was tested, with the results indicating adequate fit indexes (X^2^_(332)_ = 787.75; *p* < 0.05; RMSEA = 0.055; CFI = 0.908; NFI = 0.880; NNFI = 0.900; IFI = 0.909). Next, the three theoretical models were compared (see Table 1).

As indicated by the confidence intervals, all three models were found to be significantly different from each other, thus confirming that they are indeed different structural models. The model with the poorest adjustment was M1 (in which the four dimensions directly influence family socialization style), since, although its Root Mean Square Error of Approximation (RMSEA = 0.060) and the ratio between the Chi-squared value and the degrees of freedom (X^2^/df = 2.57), understood as general fit indicators, were within acceptable limits, the other indicators (CFI = 0.894; NFI = 0.839; NNFI = 0.880; IFI = 0.895) failed to reach the required cut-off point. Moreover, both the AIC and the CAIC had higher values than in the other two models, thereby indicating the poorest fit of all three models tested.

**Figure 2:**
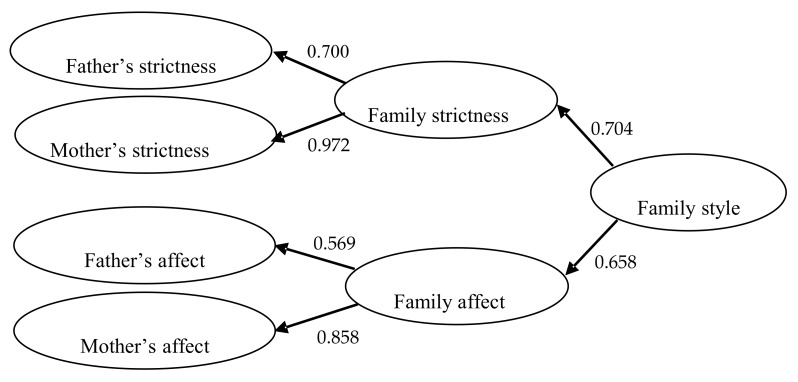
Standardized solutions of the accepted M3 model.

Both M2, which postulated that family socialization style is determined by the father’s style and the mother’s style (X^2^_(330)_ = 765.72; X^2^/gl = 2.32; p < 0.05; RMSEA = 0.054; CFI = 0.912; NFI = 0.856; NNFI = 0.900; IFI = 0.913), and M3, according to which family socialization style is the result of the family affect-communication style and family strictness style (X^2^_(330)_ = 668.51; X^2^/gl = 2.02 p < 0.05; RMSEA = 0.048; CFI = 0.932; NFI = 0.894; NNFI = 0.922; IFI = 0.932), were found to have a good fit. Nevertheless, M3 had better values, since its Chi-squared value, degrees of freedom ratio, and RMSEA were lower, and its CFI, NFI, NNFI, and IFI were higher than for M2. Moreover, although the NNFI value failed to reach the minimum 0.90 cut-off point, it did come very close to this limit. It should be remembered that the acceptance of any model is determined by the combination of all of its indicators, with experts recommending against taking any one value as a single reference for accepting or rejecting a model [67]. M3 also obtained lower AIC (AIC_(M2)_ = 105.728; AIC_(M3)_ = 8.515) and CAIC (CAIC_(M2)_ = –1579.590; AIC_(M3)_ = –1676.802) index values than M2, thus indicating that it is the preferred model for acceptance (Figure 2). In none of the cases did the indices of modification and improvement of the three models point to the need to establish correlations between the indicators of the Strictness and Affect dimensions, on the contrary, their incorporation worsens the fit of any of the models, thus pointing to the orthogonality of the two classic dimensions.

Regarding the effects observed between the variables studied, an individual analysis of the regression coefficients of the final model (Figure 2) revealed that all of the proposed pathways reached significance level (*p* < 0.01). Family socialization style was determined to a very similar extent by the general strictness style in the home (β = –0.704; p < 0.01) and the degree of affect and communication demonstrated by both parents (β = 0.658; *p* < 0.01), although strictness had a slightly greater influence (R^2^ = 0.496) than affect-communication (R^2^ = 0.433), as well as a negative effect on family socialization style.

The mother’s strictness was found to have considerably more influence (β = 0.972; *p* < 0.01; R^2^ = 0.944) than the father’s (β = 0.700; p < 0.01 R^2^ = 0.490) in determining family strictness style. The same was also true for family communication and affect style, i.e., it was the mother’s affect and communication (β = 0.858; *p* < 0.01; R^2^ = 0.735) that had a greater effect than the father’s (β = 0.569; p < 0.01; R^2^ = 0.324) on family affect-communication style, as perceived by the child.

## 4. Discussion

The present study aimed to explore the influence of parenting practices on the establishment of family socialization style, understood as an emotional context or climate [13] in which parenting practices may acquire different meaning when both the mother’s and father’s affect-communication and strictness are combined. Previous studies have often focused on analyzing the style of a single parent, mainly the mother [22,37], although subsequently the weight of both parents in adolescent socialization and development has been recognized, with some authors studying the effect of each parent’s practices on children’s adjustment or maladjustment separately [24,25,26,41,69].

Nevertheless, only a few studies have sought to explore family socialization style [25,34,53,54], despite the fact that it is a precursor to the practices of both parents and vital to determining how they interact and combine to explain final development outcomes. Indeed, the few studies that mention family style either base their observations on a single parent, ask participants for a general assessment without distinguishing between father and mother, or simply calculate the mean score obtained by both [3,47,48,49,70]. However, they do not analyze the specific contribution made by each dimension to the family socialization style, as perceived by children. Hence the decision to explore in this study the way in which the parenting practices of both parents combine in the child’s assessment of the global family socialization style.

In this study, the analysis of the proposed models indicated that family socialization style derives from both the combination of the father’s and the mother’s strictness and the combination of the father’s and the mother’s affect-communication, thereby giving rise to two dimensions that could be termed "*family affect-communication*" and *"family strictness*". The combination of these two family dimensions in turn explains the family climate or style perceived by children. In addition, the results obtained in this work also indicate that the classical dimensions derived from parental practices (strictness and affection) are orthogonal dimensions (independent of each other), which offers empirical support to the results obtained in previous research [11,12].

In light of these findings, it is important to understand the exercise of maternal and paternal socialization practices in both dimensions (affect-communication and strictness), since their combined influence gives rise to different family styles or climates. Thus, the perception of one or both parents being strict, in the absence or presence of affect-communication from the same or the other parent, or from both, will have different effects in terms of family socialization style. In other words, the most important thing seems to be the child’s global and combined perception of their parental dyad, since the mother’s and the father’s practices seem to combine in each dimension, having both a synergistic and a certain buffer effect on one another [58], depending on the type of style adopted by each. It is therefore important to infer family style on the basis of the way in which paternal and maternal dimensions relate to each other, in order to determine their combined effect [17,25,34] and the proportional weight of each, rather than just a mean score, as has been the case in the past [49].

Another conclusion that can be drawn is that the two dimensions are almost equally important for determining family style, although strictness has a somewhat greater and negative effect, with an extremely strict family style on occasions canceling out the effect of even a high level of affect and communication. The results of this study are consistent with that observed in previous studies, in which, unlike parental discipline (particularly punitive and coercive discipline), both maternal and paternal affect seem to correlate positively with certain aspects of adolescent adjustment [1,71,72]. Our findings also coincide with those reported by studies which observed better adolescent adjustment outcomes when young people are brought up with a democratic or, particularly, indulgent style, characterized by a high level of affect and the absence (or moderate level) of strict discipline [3,4,73,74]. Neglectful and authoritarian styles, on the other hand, seem to be more closely related to adolescent emotional instability, maladjustment, and violence [3,48,49]. These results question the need for strict, authoritarian rule setting, particularly during adolescence, a vital moment in young people’s lives in which they need greater support for their autonomy [25] and therefore types of discipline based on guidance and reflection provided through affect and communication [71,75].

The results of the present study offer information regarding the weight of maternal and paternal practices in the establishment of a family socialization style, with the mother playing a predominant role in both dimensions, probably because traditionally, mothers have tended to spend more time with their children than fathers [38,39]. Furthermore, despite changes in modern society, it seems that in general, women continue to assume greater responsibility for their children’s care and upbringing [21,40]. Indeed, previous research has found that adolescent children tend to award higher scores to their mother than to their father in all dimensions, both positive and negative (affect, communication, control, discipline, etc.) [2,37,55,56,57].

It has also been found that children develop different expectations regarding their parents’ behavior depending (to a large extent) on the socially-established rules for men and women existing in the surrounding culture, which would explain the different weight of paternal and maternal practices in children’s perception of family style [17,37]. In other words, it is possible that, given existing female stereotypes regarding empathy, attention to other people’s needs, and sensitivity [76], as well as the social idea regarding what it means to be a "good mother" (i.e., dedicated, exclusive and caring [77], self-denying, and offering unconditional love and support), it may be that a child’s perception of severe or strict imposition of rules by their mother goes against both the "maternal ideal" and those stereotypes associated with women, and may therefore be counterproductive to the establishment of a positive family style. Indeed, previous research supports this idea, indicating that family conflict is reduced when parents, particularly mothers, use inductive discipline [75]. For its part, the continuing stereotype of the father figure as the breadwinner and ultimate authority in the family [38] may enable men to exercise strict discipline without having such a negative effect on family style as when it is exercised by women. This is consistent with the findings reported by other studies, in which maternal control is perceived as intrusive whereas paternal control, in the same environment, is considered more legitimate [26] and is not therefore perceived so negatively and is not linked (as maternal control is) to children’s stress [78] or behavioral problems [25]. Similarly, it is possible that paternal affect may contribute less to family style than maternal affect due to the social expectation of men’s greater independence [76] and autonomy, and therefore their lesser involvement in care tasks.

It is also possible that the different weight of mothers’ and fathers’ contribution to family socialization style may be due to other issues that have not been considered in this study, such as the age and sex of the child, or the age of the parents themselves [17,26,78,79,80]. In this respect, although there are numerous studies that find a differential impact of parental styles on adolescent development according to whether it is perceived from the father or from the mother [17,25,27], there is no clear consensus on the matter, as there is also evidence of a very similar impact [81]. Therefore, the differences could be due either to the sex of the dyad analyzed [25,80], or to the sex of the parent and the type of dimension studied [17], or even to the developmental phase or age of each member of the family, or, probably, to a combination of all of them. In this sense, considering jointly the dimensions of both parents in the form of socialization family style could contribute to clarify these complex relationships. In regards to the developmental phase or age for example, mothers have been found to have more influence during childhood and early adolescence [80], while fathers seem to become more prominent figures in their children’s lives towards the end of this period [2] and during young adulthood [36]. Besides, traditionally it has been considered that with increasing age, parents adapt their parental behaviors to their children’s changing needs, which translates into a child’s perception of less control [82], support, and affection [44], especially in the case of older adolescents. Indeed, previous studies have found that the incidence of maternal dimensions decreases with age, being higher at the beginning of adolescence [78]. In any case, there is not a unique consensus about the moderating effect of age, as sometimes no differences were found, observing the protective effect of an affective parenting style regardless of children’s age [48,49]. It would be, therefore, particularly interesting for future research to consider the possible moderating effect of variables such as sex and age on the influence that the family socialization style may have on adolescent development [80,83].

Another aspect that has not been considered in the present study is parenting consistency/inconsistency and how this affects family style and, ultimately, children’s development. One specific element of this would be consistency between parents, which despite being considered vital to children’s adjustment, has received relatively little empirical attention [53]. Indeed, the few previous studies which have been carried out in this field seem to indicate better adjustment among adolescents whose parents have similar parenting styles, characterized by a predominance of affect-communication [22,33,35,84], a finding which is consistent with the results observed here, which suggest that the affect-communication of both parents makes a significant and positive contribution to family style. Nevertheless, it has yet to be confirmed whether consistency between maternal and paternal parenting practices [35,84,85,86] really does foster a family socialization style that is more positive for children’s and young people’s development, and if so, how specifically it contributes to said psycho-social adjustment. In fact, there is evidence of the buffering effect of positive parental practices (e.g., affective) of one parent when there is a low quality in the relationship with the other [58], so it would be of great interest to delve deeper into these aspects of the consistency between paternal and maternal dimensions and their combination in the family style.

Another limitation to bear in mind is linked to the measures of parenting dimensions and styles. In this case, adolescent perceptions were used to determine these variables, since previous studies consider them to be more objective and less influenced by aspects such as the social desirability bias [3,4,21]. Nevertheless, future studies may wish to complement these perspectives with “objective” reports by outsiders, such as researchers [17], or with self-perception measures administered to parents themselves. In fact, the same instrument that has been used in this study presents a version to be completed by parents [62], although there are other tools that have also been used and adapted to include the filial, paternal, and maternal perspective [87], e.g., Children’s Report of Parental Behavior Inventory (CRPBI; [88]; Psychological Control Scale [89]). Previous research seems to show that there is a significant but moderate correlation between the perceptions of children and parents, although the study of the possible divergences is pointed out as an interesting line of research; it seems to depend, in part, on both the sex of the dyad and the measured construct [87].

Finally, it is noteworthy that, although the majority of adolescents who participated in this study (84.9%) lived with their nuclear or nuclear-extended family, another challenge for future research would be to replicate the results with other types of families, such as ones in which the parents are separated or divorced. In fact, even if previous research suggests that, regardless of family structure, it is the perception of affect and support from parents which is strongly associated to children’s developmental outcomes (e.g., self-esteem) [43], there could be differences in the weight of the dimensions in regards to the sex of the parent. As a matter of fact, in some studies, in joint-custody arrangements, having a supportive father appears to be as important [90], or even more, than having a supportive mother [43]. Besides, the type of arrangement seems to be related with the practices and parenting style of both the father and the mother, with non-residential fathers being more likely to be uninvolved or permissive, as compared to co-parenting or residential fathers, who tend to show a greater coherence with the mother’s style, a style of both of them that tends to be a more affective and involved one (e.g., democratic) [91]. Taking all of this into account, future research should go into greater depth with regard to the relations and interaction between the different dimensions of the parenting styles of both separated or divorced parents, as well as how those arrangements could influence the family parenting style [91]. Step families are another type of family which it would be interesting to analyze, since here we see the emergence of new "paternal" and "maternal" figures who engage in children’s upbringing without actually being their birth parents. Also, families made up by two fathers or two mothers, who may have already gone through a process of reflection regarding the kind of role each partner should play, and between whom there is perhaps not such an unequal presence as has traditionally occurred between fathers and mothers.

Another perspective not considered in this study is the possible influence of parental or adolescent’s psychological profiles [92,93], which could affect the way children perceive their relationship with their parents, and therefore, would influence both their perception of family style and their developmental outcomes. This type of approach would move beyond the aim of the majority of studies carried out to date in this field, which have focused mainly on nuclear families and have not taken into account the psychological profile of the participants.

## 5. Conclusions

This study highlights the importance of taking both the maternal and paternal figures into consideration when studying family socialization style, since despite recent social changes, the heterosexual two-parent family makeup continues to be the majority one, at least in the sample analyzed here. Therefore, if we wish to gain a better understanding of the complex relationship between family socialization and adolescent adjustment, it is vital to gather information on the practices of both parents [24,59,60]. 

Consistent with previous studies [3,4,73,74], the results found here confirm that displays of affection and good communication between parents and adolescent children makes a more important and positive contribution to the establishment of a positive family socialization style than strict rule setting, as well as helping to reduce conflict in the home environment [75]. For its part, the strict imposition of rules appears to be counterproductive, particularly during adolescence, a vital life stage in which young people seek to establish their identity and require a greater degree of support for the development of their autonomy [25]. It was also observed that, for both affect and strictness, mothers continue to have a stronger influence than fathers do on family socialization style. This seems consistent with the fact that, despite women’s increased presence in the public sphere and on the labor market, they continue to spend more time than men on care tasks [37], particularly family-related ones. Nevertheless, in line with that reported by previous research [17], the findings of the present study also highlight the importance of the father figure in the establishment of family style, which is why it is important to involve men more in children’s upbringing and care, as well as in parenting support programs and interventions [94]. Given that children’s upbringing is influenced by both the presence and absence of certain elements, and in light of the importance of affect and communication for both the establishment of family style or climate and adolescent development and adjustment [14,15,19,20], it is vital to develop programs and actions which seek to foster and promote the knowledge and use of positive parenting practices based on communication, affect, and listening. It is also imperative to implement policies designed to disseminate the importance of fathers’ involvement in their children’s upbringing, not only during childhood but also during adolescence, a developmental stage that is especially complex [5] due to the number of challenges young people must face at the same time, and during which, despite the growing importance of peers and friends, family continues to play a key role.

## Figures and Tables

**Table 1:** Goodness-of-fit indicators for the hypothesized theoretical models.

Model	X2 (df)	*p*	X2/df	CFI	NFI	NNFI	IFI	RMSEA (90% CI)	AIC	CAIC
M1	854.53 (332)	0.000	2.57	0.894	0.839	0.880	0.895	0.060 (0.055–0.065)	190.533	−1504.999
M2	765.72 (330)	0.000	2.32	0.912	0.856	0.900	0.913	0.054 (0.049–0.059)	105.728	−1579.590
M3	668.51 (330)	0.000	2.02	0.932	0.894	0.922	0.932	0.048 (0.043–0.053)	8.515	−1676.802

Note： Comparative Fit Index (CFI), Normed Fit Index (NFI), Non-Normed Fit Index (NNFI), Incremental Fit Index (IFI), Root Mean Square Error of Approximation (RMSEA), Akaike Information Criterion (AIC) and Consistent Akaike Information Criterion (CAIC).
